# Jejunal intussusception and perforation due to enteric muco-submucosal elongated polyp: a case report and literature review

**DOI:** 10.1186/s40792-022-01584-6

**Published:** 2023-01-11

**Authors:** Ryosuke Kikuchi, Shigenobu Emoto, Hiroaki Nozawa, Kazuhito Sasaki, Koji Murono, Shinya Abe, Hirofumi Sonoda, Aya Shinozaki-Ushiku, Soichiro Ishihara

**Affiliations:** 1 Department of Surgical Oncology, The University of Tokyo Hospital, 7-3-1 Hongo, Bunkyo-Ku, Tokyo, 113-8655 Japan; 2 Department of Pathology, The University of Tokyo Hospital, 7-3-1 Hongo, Bunkyo-Ku, Tokyo, 113-8655 Japan

**Keywords:** Enteric muco-submucosal elongated polyp, Intussusception, Perforation, Surgery

## Abstract

**Background:**

A muco-submucosal elongated polyp is a non-neoplastic growth composed of mucosa and submucosa. Although muco-submucosal elongated polyps are commonly reported in the large intestine, they are rare in the small intestine, in which they are called enteric muco-submucosal elongated polyps. We herein present a case of jejunal intussusception and perforation due to an enteric muco-submucosal elongated polyp.

**Case presentation:**

A 46-year-old woman presented with abdominal pain and vomiting. Computed tomography revealed jejunal intussusception, which was reduced via a nasointestinal ileus tube. Oral double-balloon endoscopy showed an elongated polyp in the proximal jejunum. The patient refused surgical resection and thus, the polyp was monitored. Six months later, the patient was readmitted with the recurrence of jejunal intussusception and underwent emergency surgery. Intraoperative findings revealed an intussuscepted bowel with an elongated polyp and multiple perforations in the proximal jejunum. We resected approximately 90 cm of the bowel, including the intussuscepted segment and perforated sites. The pedunculated polyp, which was 60 mm in length, was located on the oral side of the resected specimen. Histopathologically, the polyp was covered by normal mucosa and the submucosa consisted of edematous loose connective tissue. The histopathological diagnosis confirmed an enteric muco-submucosal elongated polyp.

**Conclusions:**

Symptomatic patients with enteric muco-submucosal elongated polyps may be at risk of complications, as observed in the present case, and need to undergo timely resection.

## Background

A muco-submucosal elongated polyp (MSEP) is a non-neoplastic growth composed of mucosa and submucosa [1]. The characteristic endoscopic feature of MSEP is an elongated, slender, “worm-like” appearance. MSEP is commonly reported in the large intestine and is designated as a colonic muco-submucosal elongated polyp (CMSEP). However, it is rare in the small intestine and is known as an enteric muco-submucosal elongated polyp (EMSEP). We herein present a patient who underwent emergency surgery for jejunal intussusception and perforation caused by EMSEP.

## Case presentation

**Fig. 1:**
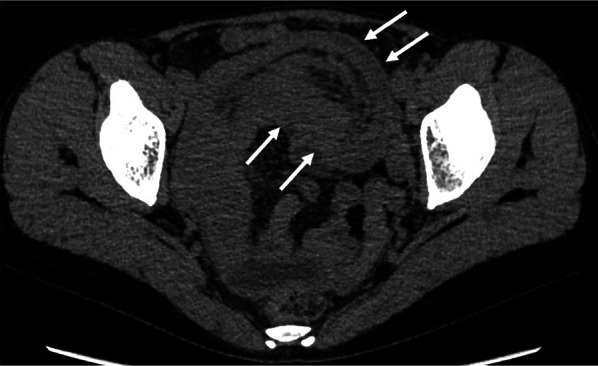
Computed tomography findings on the first admission. Computed tomography revealed intussusception (white arrows) in the jejunum

**Fig. 2:**
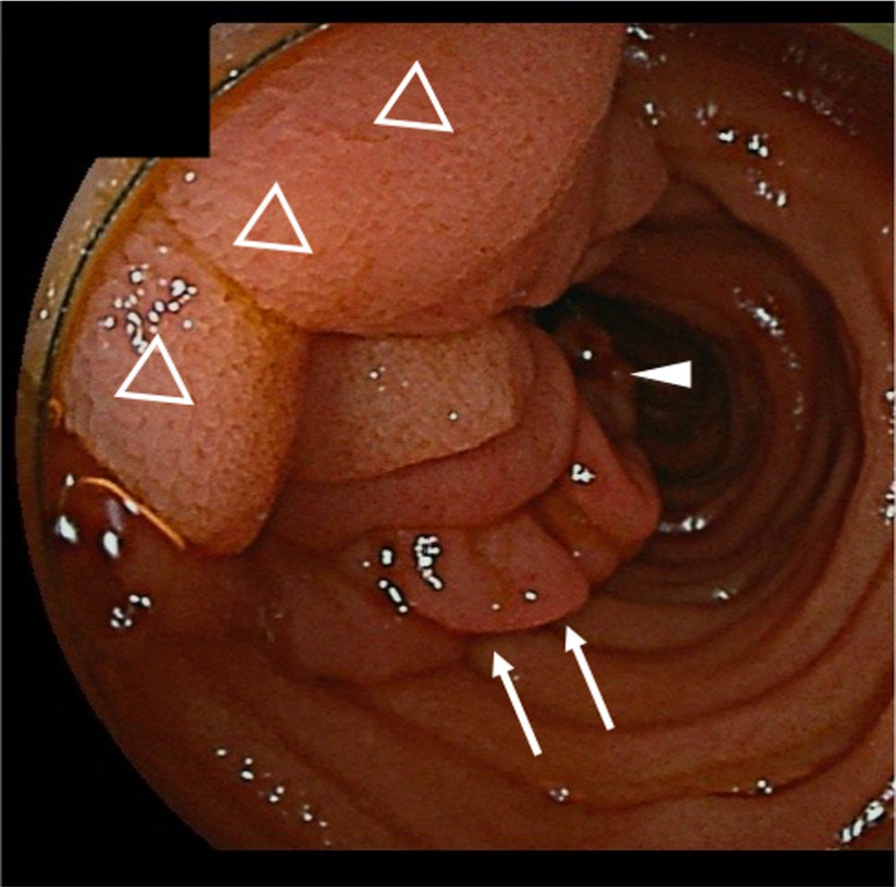
Double-balloon endoscopy findings. Double-balloon endoscopy revealed a large polyp in the proximal jejunum with a long stalk (white arrows). Closed triangle indicates the tip and open triangles show the base

**Fig. 3:**
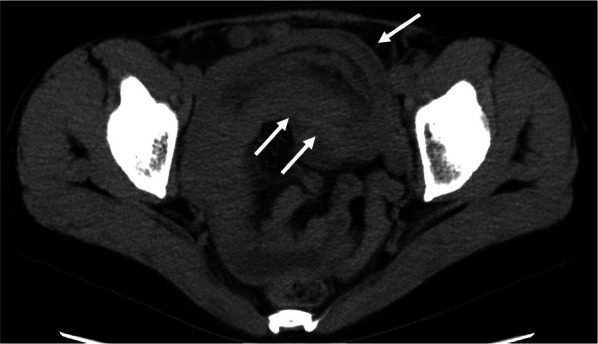
Computed tomography findings on the second admission. Computed tomography revealed the recurrence of jejunal intussusception (white arrows) without any free air or fluid

A 46-year-old woman presented to the emergency department of our hospital with abdominal pain and vomiting. She had a history of dilated cardiomyopathy and asthma and was taking a β-blocker. Upper abdominal pain had initially developed approximately 6 months earlier. Non-contrast computed tomography (CT) revealed intussusception in the jejunum (Fig. 1). However, we could not conduct a contrast-enhanced CT owing to the patient's asthma. Since the non-contrast CT did not reveal bowel wall thinning or increased fatty tissue density around the intussusception, we supposed that ischemic changes were not evident. A nasointestinal ileus tube was placed, which spontaneously reduced intussusception 2 days after admission. Gastrografin contrast examination through the nasointestinal ileus tube demonstrated dilation of the small bowel on the oral side of the obstruction, and a small amount of Gastrografin flowed to the anal side. No characteristic findings of intussusception, such as crab’s claw-like appearance, were observed. Oral double-balloon endoscopy revealed a solitary, elongated polyp in the proximal jejunum (Fig. 2). Biopsy indicated chronic inflammation without neoplastic changes. Endoscopic treatment was considered impossible because of the inability to observe the entire polyp. Surgical resection was scheduled, and we tattooed around the polyp for indication. However, the patient refused surgical resection. Therefore, she was discharged and the polyp was monitored. Six months later, the patient was readmitted to our hospital with abdominal pain and vomiting. The patient’s vital signs were as follows: blood pressure, 120/68 mmHg; pulse, 110 beats per minute; and body temperature, 37.0 ℃. Her abdomen was distended and tender in the upper abdominal region, but Blumberg’s sign and muscular defense were absent. The findings of laboratory examinations revealed a white blood cell count of 8200/μL (neutrophils, 86%), an elevated C-reactive protein level (8.0 mg/dL), and a reduced serum albumin level (2.5 g/L). CT findings on the second admission revealed the recurrence of jejunal intussusception without any free air or fluid and were almost identical to the findings observed on the initial admission (Fig. 3). The patient still refused to undergo surgery. However, her abdominal pain worsened, so that emergency surgery was performed.

**Fig. 4:**
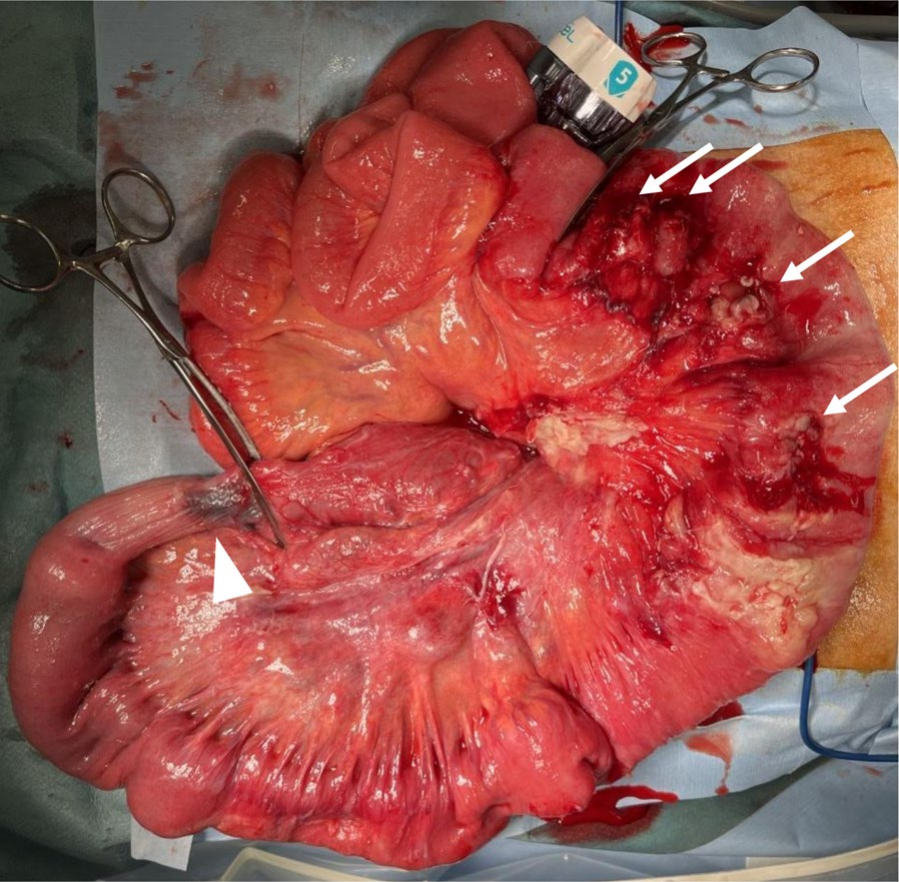
Intraoperative findings after reducing intussusception. The tattoo around the polyp was located on the oral side of the intussuscepted segment (closed triangle) and multiple perforations were on the anal side (white arrows)

**Fig. 5:**
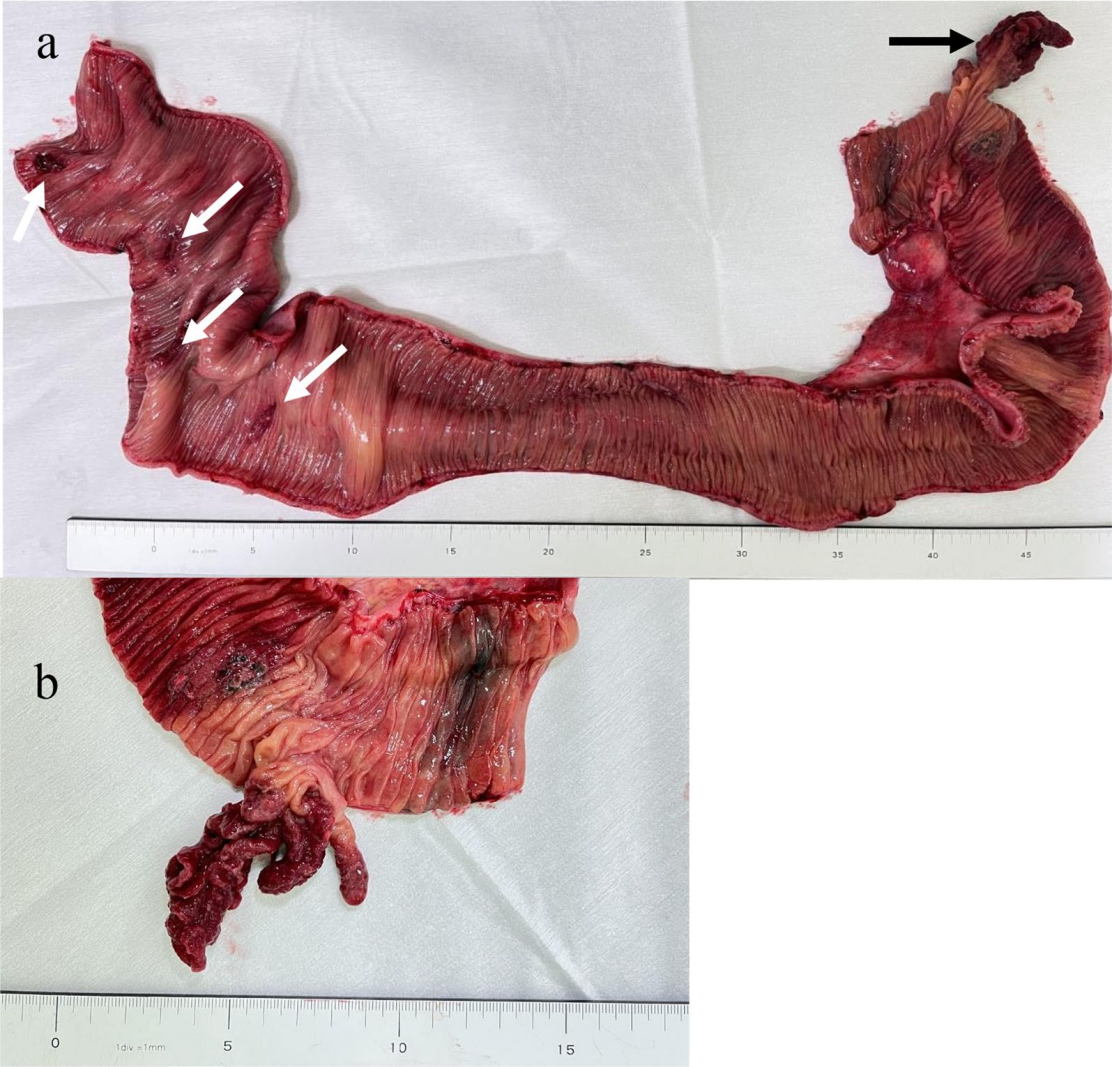
Macroscopic appearance of the surgically resected specimen. **a** The polyp was located on the oral side of the resected specimen (black arrow) and four perforated sites were on the anal side (white arrows). **b**There was a pedunculated polyp of 60 × 24 mm in size

**Fig. 6:**
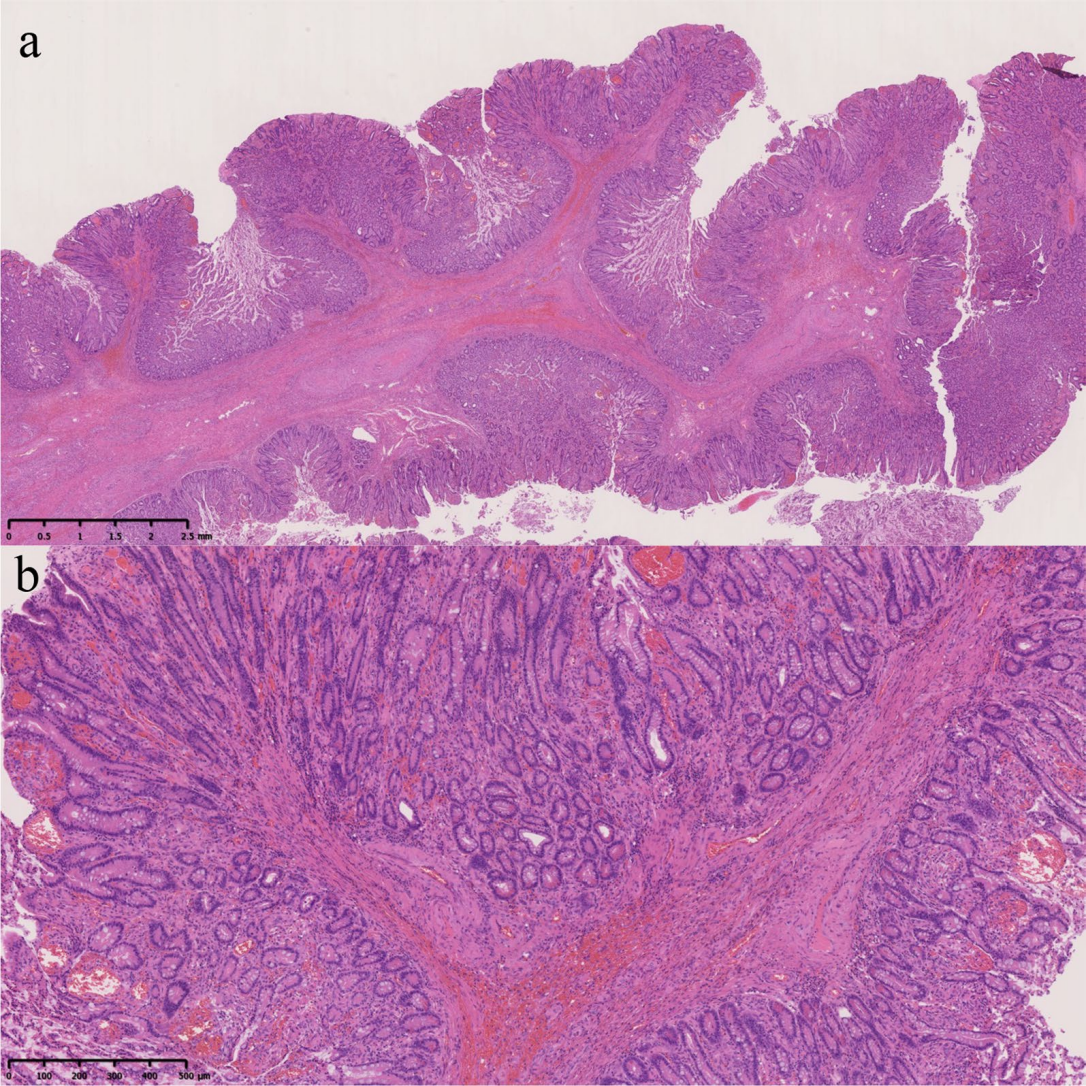
Histopathological findings. **a **Hematoxylin–eosin staining (original magnification: × 20) showing the polyp covered by normal mucosa and submucosa. **b **Hematoxylin–eosin staining (original magnification: × 100) showing the submucosa containing edematous loose connective tissue with prominent vascular and lymphatic components

Surgery was initially performed laparoscopically. A 12-mm port was placed in the abdominal cavity through an umbilical incision, and an additional 5-mm port was inserted in the right lower quadrant. We observed a moderate amount of cloudy fluid and an intussuscepted bowel loop just under the umbilical incision. Since intussusception was difficult to reduce laparoscopically, we moved to laparotomy. We initially reduced intussusception manually by pulling the bowel loop. There were four perforated sites in the intussuscepted segment of the proximal jejunum (Fig. 4). We resected a 90-cm bowel segment, including the intussuscepted segment and perforated sites, and performed a functional end-to-end anastomosis. A pedunculated polyp measuring 60 mm × 24 mm was present on the oral side of the resected specimen and the perforated sites were on the anal side (Fig. 5). Histopathological findings showed that the polyp was covered by normal mucosa, and the submucosa consisted of edematous loose connective tissue with prominent vascular and lymphatic components and no neoplastic changes (Fig. 6). The histopathological diagnosis was EMSEP. Moreover, histopathological examination revealed multiple ulcerations around the perforated sites. These multiple ulcerations were caused by ischemic changes as a result of jejunal intussusception. Thus, the multiple perforations were caused by jejunal intussusception.

The patient recovered well without any postoperative complications. She was able to take a clear liquid diet on postoperative day 4 and was discharged on postoperative day 12. Seven months later, her nutritional status had recovered, and the symptom of chronic abdominal pain disappeared postoperatively.

## Discussion

We herein reported a rare case of jejunal intussusception and perforation due to EMSEP. MSEP is relatively rare in the small intestine, but is more common in the large intestine. Histologically, MSEP is composed of normal mucosa and a submucosal layer containing dilated lymphovascular components with edematous connective tissue [1]. In 1998, Matake et al. reported 15 cases of CMSEP as elongated non-neoplastic polyps comprising mucosa and submucosa [1]. CMSEP is estimated to account for only 0.1% of all colorectal polyps [2] and the majority of CMSEP cases have been reported in Asia, particularly in Japan [1]. Ezoe et al. hypothesized that continuous peristalsis with mechanical irritation elevates the submucosal layer, leading to polyp elongation [3]. The characteristic endoscopic feature of MSEP is an elongated, slender, “worm-like” appearance [4]. Although one study suggested a relationship between diverticulosis and CMSEP [5] and another proposed the familial occurrence of EMSEP [6], the etiopathogenesis remains unclear in the present case.

There are only seven documented case reports of EMSEP on the PUBMED database, which are shown in addition to the present case in Table 1 [3, 4, 6–10]. Seven polyps were located in the duodenum and five in the proximal jejunum. No polyps were observed in the ileum. Five polyps in the proximal jejunum were detected via oral double-balloon or capsule endoscopy. Although the most common symptom of EMSEP in the proximal jejunum is melena, our patient initially developed upper abdominal pain approximately 6 months before presentation; the symptom of this polyp was considered to be abdominal pain. Ten polyps were treated endoscopically, and surgical resection was performed on the remaining two patients. Only a single case of CMSEP with intussusception has been reported to date [11], and to the best of our knowledge, this is the first case report of EMSEP associated with intussusception.

**Table 1:** Literature review for EMSEP

First author (reference)	Year	Age (year)	Sex	Location	Size (mm)	Symptoms	Therapy
Ezoe (3)	2003	72	F	Duodenum	48	None	Endoscopic treatment
Ezoe (3)	2003	56	F	Duodenum	17	None	Endoscopic treatment
Ezoe (3)	2003	70	F	Duodenum	17	None	Endoscopic treatment
Sugimori (7)	2008	53	M	Proximal jejunum	118	Melena	Endoscopic treatment
Kim (8)	2010	58	F	Duodenum	50	Postprandial discomfort	Endoscopic treatment
Nishimura (9)	2012	70	F	Duodenum	70	Melena	Endoscopic treatment
Tan (4)	2013	55	F	Duodenum	40	Postprandial discomfort	Endoscopic treatment
Tan (4)	2013	70	M	Duodenum	22	Reflux symptom	Endoscopic treatment
Shimamura (6)	2016	67	M	Proximal jejunum	32	Melena	Endoscopic treatment
Shimamura (6)	2016	64	M	Proximal jejunum	20	Melena	Endoscopic treatment
Okamura (10)	2020	78	F	Proximal jejunum	125	Melena	Surgical resection
Our case	2022	46	F	Proximal jejunum	60	Abdominal pain	Surgical resection

*EMSEP* enteric muco-submucosal elongated polyp, *F* female, *M* male

In contrast to that in children, intussusception in adults is relatively rare and commonly occurs with a pathological leading point [12]. Regarding the treatment of adult intussusception, whereas the conventional treatment of choice was surgical resection, recent studies showed good outcomes for conservative treatment [13]. On the other hand, we need to consider surgical intervention especially in cases of acute abdominal pain [12]. In the present case, EMSEP was the cause of jejunal intussusception and surgical resection was performed when abdominal pain worsened due to the recurrence of jejunal intussusception. Therefore, we suggest that symptomatic patients with known EMSEP need to undergo prompt resection. Furthermore, although CT did not show any free air or fluid, intraoperative findings revealed multiple perforations. CT findings in patients with intussusception may be atypical. In contrast, an ultrasound examination may aid the diagnosis of intussusception and perforation. Unfortunately, we did not conduct an ultrasound examination.

There is currently no established treatment strategy for asymptomatic EMSEP and a potential relationship between EMSEP and neoplasms has not been proposed. A previous study suggested a relationship between CMSEP and neoplasms [14]: they reported a small area of adenocarcinoma invading the submucosal layer on the tip of CMSEP and indicated that long-term mechanical stress promoted the growth of the existing tumor. Based on a potentially similar etiology between EMSEP and CMSEP, EMSEP may also be associated with neoplasms. Moreover, since EMSEP is covered by normal mucosa, it is difficult to distinguish it from a submucosal tumor with superficial biopsies. Thus, resection may be warranted for patients with asymptomatic EMSEP; however, more cases are needed to confirm this recommendation.

## Conclusions

We encountered a case requiring surgical resection of an extensive small bowel loop because of intussusception and perforation due to EMSEP. This rare case suggests that symptomatic patients with known EMSEP need to undergo prompt resection.

## Data Availability

The datasets of this case report are available from the corresponding author upon reasonable request.

## Abbreviations

MSEP: Muco-submucosal elongated polyp
CMSEP: Colonic muco-submucosal elongated polyp
EMSEP: Enteric muco-submucosal elongated polyp
CT: Computed tomography
